# Prolonged Terlipressin Therapy as a Bridge to Curative TIPS for HRS–AKI in a Nontransplant Candidate

**DOI:** 10.1155/crhe/5364905

**Published:** 2026-02-27

**Authors:** Deirdre Reidy, Arun Jesudian, David Salerno, Catherine Lucero

**Affiliations:** ^1^ Department of Internal Medicine, Weill Cornell Medicine, New York, 10021, New York, USA, cornell.edu; ^2^ Division of Gastroenterology and Hepatology, Weill Cornell Medicine, New York, 10065, New York, USA, cornell.edu; ^3^ Department of Pharmacy, NewYork-Presbyterian Hospital, New York, 10021, New York, USA

## Abstract

Hepatorenal syndrome–acute kidney injury (HRS–AKI) is a severe complication of advanced cirrhosis with high mortality and limited treatment options. Terlipressin is currently the only FDA‐approved therapy for HRS, though recurrence is common upon discontinuation in patients without access to liver transplantation. Transjugular intrahepatic portosystemic shunt (TIPS) may provide definitive treatment for HRS–AKI in nontransplant candidates or in the setting of prolonged waitlists. This case illustrates the safety and success of terlipressin prior to TIPS for definitive treatment of HRS–AKI in a nontransplant candidate.

## 1. Introduction

Hepatorenal syndrome–acute kidney injury (HRS–AKI) is a type of AKI that occurs in patients with advanced cirrhosis and portal hypertension, caused by renal vasoconstriction in the setting of systemic and splanchnic vasodilation [1]. Prognosis of HRS–AKI is poor, with estimated life expectancy of days to weeks if left untreated [2, 3]. Terlipressin, a vasopressin analog, has emerged as the primary pharmacologic treatment for HRS [4]. It is typically administered for up to 14 days and usually discontinued earlier if there is no response after 3–4 days or 24 h after creatinine decreases below 1.5 mg/dL [4]. While liver transplant (LT) remains the only definitive treatment for HRS–AKI and cirrhosis, many individuals are not LT candidates or do not undergo timely LT due to long waiting time and limited organ availability. In such cases, transjugular intrahepatic portosystemic shunt (TIPS) placement has been explored as an alternative strategy to reduce portal pressures and improve renal function [5–8]. Here, we present a case of recurrent HRS–AKI requiring prolonged terlipressin therapy that was successfully bridged to TIPS in a nontransplant candidate.

## 2. Case

A 63‐year‐old female with Type 2 diabetes mellitus and cirrhosis secondary to metabolic dysfunction–associated steatohepatitis (MASH) complicated by refractory ascites and nonbleeding esophageal varices presented to the hospital with worsening ascites. She had previously been evaluated for LT but was not listed due to lack of caregiver support. The patient was adherent to her home medications, including insulin, carvedilol 6.25 mg twice a day, furosemide 20 mg every other day, spironolactone 25 mg daily, and rifaximin 550 mg twice a day.

**FIGURE 1:**
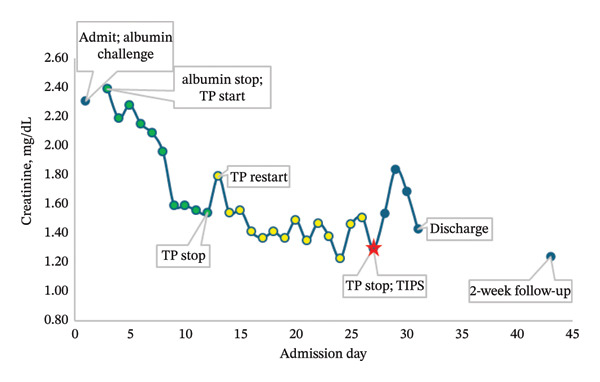
Creatinine trend on terlipressin (TP) and following TIPS for HRS–AKI.

Upon admission, she was identified to have a new AKI. Her serum creatinine level on admission was 2.31 mg/dL, increased from her baseline of 1.2‐1.3 mg/dL Furosemide and carvedilol were held on admission. Infectious workup, including blood cultures, urinalysis, urine cultures, and diagnostic paracentesis, was negative. Renal ultrasound was negative for obstruction or hydronephrosis. Abdominal ultrasound showed large volume ascites, as well as patent portal and hepatic veins. Urine electrolytes were collected with fractional excretion of urea of 1.7%, suggesting a prerenal etiology. The patient was started on an albumin challenge on the day of admission and received 75 g (1 g of albumin per 1 kg body weight) of 25% albumin on Days 1 and 2. Following the albumin challenge, creatinine increased to 2.39 mg/dL. The patient was started on IV terlipressin 0.85 mg every 6 h with progressive improvement in creatinine (Figure 1). Creatinine was 2.15 mg/dL on Day 4 of treatment, and the patient was continued on the same dose and frequency. Creatinine improvement plateaued to 1.54 mg/dL over 8 days, at which point terlipressin was discontinued. Treatment with midodrine was deferred as systolic and mean arterial pressures were normal. The following day, creatinine increased to 1.79 mg/dL, and terlipressin 0.85 mg IV every 6 h was resumed.

The patient was continued on terlipressin while TIPS was considered as a destination therapy as she was not a candidate for LT at the time. The patient remained on terlipressin for an additional 15 days while undergoing preoperative testing and evaluation for TIPS. Hepatic encephalopathy was absent prior to TIPS. Cardiac function was assessed prior to the procedure, demonstrating an ejection fraction of 55%–60%, normal left and right ventricular function with borderline‐to‐mild pulmonary hypertension (right ventricular systolic pressure = 29.7 mmHg). MELD‐Cr score at TIPS was 16. The portosystemic gradient was reduced from 24 to 4 mmHg post‐TIPS. Mean right atrial pressure pre‐ and post‐TIPS was 19 and 22 mmHg, respectively. The patient remained admitted for 4 days, and creatinine decreased to 1.43 mg/dL at the time of discharge and to 1.24 mg/dL at 2 weeks following discharge. At discharge follow‐up, the patient remained euvolemic.

## 3. Discussion

This case highlights the potential role of terlipressin as a bridge to TIPS for treatment of HRS–AKI. However, given the rapid recurrence of HRS–AKI upon discontinuation of terlipressin therapy and the lack of LT as a curative option, this patient required TIPS insertion as a definitive treatment of her severe underlying portal hypertension which predisposed her to HRS–AKI.

The proposed mechanism of utilizing TIPS for treatment of HRS–AKI relies on redistribution of blood volume, reduction of portal pressures, and amelioration of splanchnic vasodilation through TIPS, resulting in improvement in renal blood flow. Previous studies examining the use of TIPS in HRS–AKI showed improvements in serum creatinine, glomerular filtration rates, renal plasma flow, and plasma aldosterone levels, though these studies were small, uncontrolled, or lacked long‐term follow‐up [5–9]. As a result, the 2021 AASLD guidelines on HRS do not recommend TIPS in patients with HRS–AKI, citing “insufficient information” to support use in that context [1, 10, 11].

Additionally, AKI is considered a relative contraindication to TIPS owing to concern for risk of contrast‐induced nephropathy, volume overload in the setting of major hemodynamic shifts, and worsening cardiac and liver function [12–14]. The use of terlipressin for treating HRS–AKI prior to TIPS can potentially mitigate these relative contraindications. Terlipressin is recommended for use in HRS–AKI as a bridge to LT in eligible individuals, as it improves renal function and may delay the need for renal replacement therapy, thereby potentially allowing more time for transplant evaluation and organ allocation to occur [15]. Terlipressin may be able to play a similar role in patients who are undergoing TIPS evaluation and placement, aiding in stabilization of renal function and improvement in hemodynamics prior to insertion. The novel ongoing Liver‐HERO randomized controlled trial is directly comparing TIPS to standardized care (terlipressin and albumin) in HRS–AKI, where patients randomized to the TIPS treatment arm will receive terlipressin and albumin until the procedure [5].

Current recommendations for the primary uses of terlipressin—direct treatment of HRS and in some cases a bridge to LT—are for a single course not to exceed 14 days. However, some patients may require multiple courses for recurrent HRS–AKI [4, 15]. Extended and consecutive treatment courses could be considered in scenarios where patients tolerate therapy without complications and where additional time is required to attain a clinical treatment goal.

Our patient’s case suggests that certain patients may derive benefit from a longer duration of terlipressin treatment and/or multiple courses in the setting of relapses of HRS–AKI. The patient received a total of 23 days of terlipressin, consisting of two courses lasting 8 days and 15 days, respectively, in order to safely undergo TIPS. This surpassed the 14‐day maximum treatment duration that is specified in the product label; clinical trial data establishing terlipressin’s safety and efficacy evaluated treatment courses up to 14 days, and there are limited data examining outcomes with prolonged treatment beyond this duration [4, 15]. The rationale behind a prolonged course in a case such as this is to stabilize kidney function and ensure circulatory stability prior to curative therapy. Similarly, the safety of long‐term continuous infusions of terlipressin for up to 12 weeks has been studied in certain patient populations with promising outcomes [16, 17]. Importantly, the patient was closely monitored for complications and adverse effects during the prolonged course and was able to safely undergo TIPS insertion.

While terlipressin as a bridge to TIPS for treatment of HRS–AKI may be a promising intervention, it should only be considered in a highly selective patient population with preserved hepatic function and without hepatic encephalopathy, right heart failure, or pulmonary disease [5, 11, 15]. In this context, the role of terlipressin is to optimize renal and circulatory status prior to TIPS, as TIPS placement in unstable patients carries significant risk [18].

## 4. Conclusion

In summary, our case demonstrates that an extended course of terlipressin can be utilized as bridging therapy to facilitate TIPS as a curative therapy for recurrent HRS–AKI, particularly in nontransplant candidates or those with low MELD and poor access to LT.

## Funding

This work was supported by Mallinckrodt Pharmaceuticals with grant number MWG‐HEP‐4068.

## Conflicts of Interest

These authors disclose the following: Arun Jesudian is a speaker and consultant for Mallinckrodt, Salix, and Madrigal pharmaceuticals. David Salerno serves on Mallinckrodt speakers’ bureau and advisory boards. The other authors declare no conflicts of interest.

## Data Availability

The data that support the findings of this study are available from the corresponding author upon reasonable request.
